# Cloning, characterization, and expression analysis of the *CHITINASE* gene family in *Helice tientsinensis*

**DOI:** 10.7717/peerj.15045

**Published:** 2023-03-14

**Authors:** Lulu Chen, Yuyan Hua, Wenxuan Ji, Jiayu Wang, Hua Zhao, Zhengfei Wang

**Affiliations:** Jiangsu Key Laboratory for Bioresources of Saline Soils, Jiangsu Synthetic Innovation Center for Coastal Bio-Agriculture, Jiangsu Provincial Key Laboratory of Coastal Wetland Bioresources and Environmental Protection, School of Wetlands, Yancheng Teachers University, Yancheng, Jiangsu, China

**Keywords:** Chitinase, *Helice tientsinensis*, Gene cloning, *Vibrio parahaemolyticus*, Expression analysis

## Abstract

Chitinase is a kind of glycoside hydrolase which is widely distributed in nature and encoded by multiple genes to catalyze the decomposition of chitin, which plays an important role in the molting and pathogen defense of crustaceans. However, the research on chitinase in crustaceans is mainly focused on a few species with economic value. In this study, full-length cDNA sequences of the *HtCHT1*, *HtCHT3* and *HtCHT4* genes were cloned from the mudflat crab *Helice tientsinensis* by RACE, and the sequences were analyzed. The results showed that the full-length 2,229 bp of *HtCHT1* gene encoded 627 amino acids, while the full-length 2,191 bp of *HtCHT3* gene produced 489 amino acids, and the full-length 3,312 bp of *HtCHT4* gene encoded 664 amino acids. Bioinformatics analysis showed that all the obtained chitinase proteins had the glycosyl hydrolase family 18 (GH18) catalytic domain and chitin-binding domain (ChtBD2), furthermore, HtCHT1 and HtCHT4 proteins had signal peptide domains at N-terminal. Phylogenetic analysis showed that different types of chitinase were clustered, and HtCHTs were closely related to chitinases in the *Eriocheir sinensis*. Expression profile analysis showed that the *HtCHT1*, *HtCHT3* and* HtCHT4* were significantly expressed in hepatopancreas. Furthermore, the expression of three genes was significantly up-regulated in hepatopancreas after the *Vibrio parahaemolyticus* challenge. These results suggested that *HtCHT1*, *HtCHT3* and* HtCHT4* were belonged to the *CHITINASE* gene family in *H. tientsinensis* and were potentially involved in the antibacterial immune response. This study provides essential information for further research of chitinase in *H. tientsinensis* and even crustaceans.

## Introduction

Chitin is a *β*-1, 4-linked polymer of N-acetyl-D-glucosamine (GlcNAc) and is the second rich polysaccharide biopolymer found in nature after cellulose (Park, Kwak & Kwak, 2022). The annual output of chitin is about 10^11^ tons (Chaudhary et al., 2020). Chitin is not only a vital component of the cell wall of pathogenic fungi, but also an indispensable structural component in the shells of crustaceans, exoskeletons and intestines of insects, and it forms the structural framework of partial protozoa and threadworm eggs (Oyeleye & Normi , 2018). Chitinase proteins are glycoside hydrolases (GH), which can degrade chitin into GlcNAc polymers with low molecular weight. Chitinase proteins are widely distributed and play an important role in various organisms (Beier & Bertilsson, 2013; Hartl, Zach & Seidl-Seiboth, 2012; Song et al., 2020). Chitinase proteins play critical physiological functions in crustaceans, including the degradation of the exoskeleton, the molting growth of crustaceans (Huang et al., 2010; Proespraiwong, Tassanakajon & Rimphanitchayakit, 2010), participation in the digestion of chitin food in the peritrophic membrane (Salma et al., 2012; Zhang et al., 2014), and using as immune gene participating in pathogen immunity (Tan, Degnan & Lehnert, 2000; Zhou et al., 2018).

Chitinase proteins have some specific domains, including the signal peptides, chitin-binding domain (CBD), GH18 family catalytic domain, and junction regions rich in serine (Ser)/threonine (Thr) in crustaceans. Each of the GH18 family catalytic domain contained four highly conserved motifs: motif I (KXXXAXGGW), motif II (FXGDXDWEYP), motif III (MXYDXXG) and motif IV (GXXXWXXDXD) (Lacombe-Harvey, Brzezinski & Beaulieu, 2018; Zhou et al., 2018; Salma et al., 2012). There were six cysteine residues which form three disulfide bridges in CBD, to maintain the structural stability of chitinases.

The expression of *CHITINASE* genes family of crustaceans is tissue-specific. In the three members of *CHITINASE* genes family of *Penaeus japonicus* (*Pjchi-1*, *Pjchi-2*, *Pjchi-3*), the *Pjchi-1* and *Pjchi-3* were expressed in hepatopancreas, and there was no significant difference between intermolting and pre-molting stage, while the *Pjchi-2* was expressed in stratum corneum. It is speculated that the *Pjchi-2* might play a role in molting, and the *Pjchi-1* and *Pjchi-3* might be involved in the digestion of foods containing chitin (Watanabe et al., 1996; Watanabe & Kono, 1997; Watanabe et al., 1998).

The study in *Litopenaeus vannamei* showed that the *LvCht1*, *LvCht3* and *LvCht4* were mainly expressed in hepatopancreas, while the expression of the *LvCht2* was relatively high in the eye stalk. The *LvChid1* was expressed in all tissues, with the highest expression in the hepatopancreas (Huang et al., 2010). The *LvCht5* gene was also expressed in all tissues, with the highest expression in the muscle, moreover, Niu et al. (2018) found that *LvCht5* gene was expressed highest in the heart (Niu et al., 2018). The *LvCht6* was mainly expressed in the stratum corneum, gill and eye stalk (Huang et al., 2010).

Li et al. (2015) found that *EsCHT2* showed the tissue-specific expression in the epidermis and eye stalk. *EsCHT1*, *EsCHT3*, *EsCHT4* and *EsCHT6* were all expressed in the hepatopancreas and intestine. Furthermore, *EsCHT3* was also expressed in the stomach and haemocyte, while *EsCHT4* and *EsCHT6* were expressed in the gill, muscle and stomach (Li et al., 2015).

By analyzing the expression profiles of *CHITINASE* genes in different tissues of *Scylla paramamosain* (*SpCht1*-*SpCht7*), it had found that the *SpCht1*, *SpCht3* and *SpCht4* were highly expressed in hepatopancreas. However, the *SpCht2* was only highly expressed in the eyestalk, which suggested that it might perceive environmental changes and participate in the molting process. The expression level of the *SpCht5* and *SpCht6* in digestive tract (including stomach and intestine) was the highest, suggesting that they could contribute to the reversal of peritrophic membrane and the digestion of chitin food. The *SpCht7* in *S. paramamosain* had the highest expression in the stomach, and it was speculated that the *SpCht7* might have the similar function as the *SpCht2* and *SpCht6* (Zhou et al., 2018).

The above results suggested that chitinase in crustaceans was involved in various life activities, including molting and digestion, but they exhibited different tissue-specific expression of different genes, and it might be related to the functional differentiation and coordination of family genes.

At present, *CHITINASE* genes have been widely studied in microorganisms, plants and insects, but it is relatively rare in crustaceans, moreover, it is only concentrated in some cultured varieties with high economic value (Song et al., 2020). The mudflat crab *Helice tientsinensis* is an important economic crab in coastal mud flat, because of its delicious taste. *H. tientsinensis* belongs to the Varunidae family of Decapoda, which is widely distributed throughout the mudflats of the East Sea, the Yellow Sea and the Bohai Sea in China (Wang et al., 2019). *H. tientsinensis* is completely obtained from the coastal mud flat, that is differ from other cultured commercially crabs. The study of the *CHITINASE* gene family of the mudflat crab will be helpful to understand the evolutionary relationship of the *CHITINASE* in crab. However, there are only scattered reports on feeding habits (Lan, Zhang & Yi, 2020; Huang et al., 2019) and environmental tolerance of *H. tientsinensis* (Zuo et al., 2022; Wang et al., 2019), and the study of chitinase has not been reported. In this study, *H. tientsinensis*, the representative wild species in coastal intertidal zone, was used as a template to clone the full-length cDNA sequences of the *CHITINASE* genes using rapid amplification of cDNA ends technology. Further chitinase sequence alignment and functional domain analysis were taken by bioinformatics analysis. The expression characteristics of *CHITINASE* genes in different tissues and the response patterns under *Vibrio parahaemolyticus* challenge were conducted using qRT-PCR. The purpose of this study is to reveal the functions of different *CHITINASE* genes and provide new insights into the origin and evolution of chitinases proteins not only in crabs but also in crustaceans.

## Materials & Methods

### Experimental materials

*H. tientsinensis* samples and nearby soil as well as water samples were excavated from coastal beach areas (33°13′38.62″N, 120°49′58.59″) of Dafeng Port in Yancheng, Jiangsu Province, China. *H. tientsinensis* was temporarily cultured in the laboratory at 25 °C. During the period of temporary cultivation, an appropriate amount of mineral water was added to the plastic box every day to keep the soil moist, and the dead samples were removed in time.

### RNA extraction and cDNA library construction

Hepatopancreas, gill, muscle, heart, stomach and eyestalk tissues/organs of *H. tientsinensis* were ground to powder under liquid nitrogen. 0.02 g powder was used to extract RNA from all tissues using RNA prep Pure Animal Tissue Total RNA Extraction Kit (TIANGEN, China). According to the instructions of PrimeScript™ Master Mix (Takara Bio Inc., Shiga, Japan), cDNA libraries of different tissues were constructed.

### Cloning of conserved sequence of cDNA

cDNA library of the hepatopancreas in *H. tientsinensis* was used as a template, and primers were designed to clone the conserved region of *CHITINASE* genes according to the RNA sequencing results and some known *CHITINASE* gene homology sequences from the shrimp and crab of Decapoda in NCBI GenBank (Table S1). The PCR system is as follows: 12.5 µL PCR MIX (Takara Bio Inc., Shiga, Japan), 2 µL cDNA template (50 times diluted from cDNA library), 1 µL forward primer (10 µmol/L), 1 µL reverse primer (10 µmol/L) and adding ddH_2_O to 25 µL. The PCR mixture was put into the PCR instrument (Eppendorf, Hamburg, Germany), and the reaction parameters were: pre-denaturation at 95 °C for 3 min; denaturation at 95 °C for 30 s, annealing at 55 °C for 30 s, elongation at 72 °C for 1 kb/1 min, 35 cycles; final extending at 72 °C for 10 min. PCR bands were separated by 1% agarose gel electrophoresis, and the gel was cut under TGreen Gel Cutter (Tiangen, China). The gel was purified according to the instructions of AxyPrep™ DNA Gel Extraction Kit (Axygen, Union City, CA, USA).

The target fragment was introduced into the pMD19T vector and connected at 16 °C for 2 h. The ligating system was as follows: 2.5 µL ligase buffer, 0.5 µL pMD19T Simple vector (Takara Bio Inc., Shiga, Japan) and 2 µL gel for DNA recovery. The ligation products were transformed into DH5 *α* competent cells by heat shock method, and appropriate amount of bacterial liquid was pipetted and evenly coated on LB carbenicillin resistance solid medium (10 g peptone, 5 g yeast extract, 10 g NaCl and 15 g Agar dissolved in 1 L ddH_2_O). After sterilization at 121 °C for 21 min, carbenicillin antibiotic with final concentration of 100 µg/L was added. Monoclone was selected and cultured with 700 µL LB liquid medium (carbenicillin resistance, 100 mg/L) at 250 rpm for 3 h at 37 °C for further PCR detection. The positive clones were sent to Shanghai Boshang Biotechnology Company (Shanghai, China) for sequencing.

### Cloning of *CHITINASE* cDNA from 5′ to 3′ ends RACE

High quality of hepatopancreas RNA in *H. tientsinensis* was used as a template, following the instructions for SMARTer^®^ RACE 5′/3′Kit (Clotech, Palo Alto, CA, USA) and cDNA libraries were constructed for RACE 5′and 3′ ends of *H. tientsinensis*. Gene specific primer (GSP) which is based on known sequences of conserved regions (Table 1) was designed for RACE and cDNA ends were cloned follow the user manual. PCR bands were separated by 1% agarose gel electrophoresis, and the gel was cut under TGreen Gel Cutter. The gel was recovered according to the instructions of AxyPrep™ DNA Gel Extraction Kit (Axygen). The gel recovery products obtained by RACE were connected to the pJET vector according to the instructions of CloneJET™ PCR Cloning Kit, and ligation products were incubated at 22 °C for 30 min. The connection system is as follows: 2.5 µL 2 × Reaction buffer, 0.25 µL pJET vector, 0.25 µL T4 DNA ligase (Thermo Fisher, Waltham, MA, USA) and 2 µL gel recovery product of DNA. The ligation products were transformed into competent DH5 *α* cells and identified by bacterial liquid PCR. The positive clones were selected and sent to Shanghai Boshang Biotechnology Company (Shanghai, China) for sequencing.

**Table 1:** Primers used in this experiment.

**Primer name**	**Primer sequence (5′–3′)**	**Application**
*CHT1*-F	ACGCATCTCATCTACACCTTCTG	*HtCHT1* mRNA conserved region cloning
*CHT1*-R	TGGTGGTGGTGGTGGACTT	
*CHT3*-F	GCGAGTGCGGCCACCAGAAAG	*HtCHT1* mRNA conserved region cloning
*CHT3*-R	GTGATGTCGGTGCCAGTGAAGGT	
*CHT4*-F	CTTCATCAACAGTGCCATTGC	*HtCHT1* mRNA conserved region cloning
*CHT4*-R	ACACAATCCGTTGCCTCCT	
*CHT1-5* RACE-R	GATTACGCCAAGCTTGGTCATCAAGTGTATGGCATCCAGTAGGCT	*HtCHT1* mRNA 5′ end cloning
*CHT1-3* RACE-F	GATTACGCCAAGCTTCGGTTCTCTACGAGGGGATGAAGGACT	*HtCHT1* mRNA 3′ end cloning
*CHT3-5* RACE-R	GATTACGCCAAGCTTTGGCGTACAGAGCCTTGGTCGTCAC	*HtCHT3* mRNA 5′ end cloning
*CHT3-3* RACE-F	GATTACGCCAAGCTTGACGGTGGAATGCCTGCCGAGAAG	*HtCHT3* mRNA 3′ end cloning
*CHT4-5* RACE-R	GATTACGCCAAGCTTGGTCTTTCTTTTGGCTGGGTCTGCTG	*HtCHT4* mRNA 5′ end cloning
*CHT4-3* RACE-F	GATTACGCCAAGCTTCGGGGACTCTGTCTACTTGAAGGCTCGT	*HtCHT4* mRNA 3′ end cloning
*qCHT1-* F	AGTTGCTGACGGACTACGGTTTCG	*HtCHT1* quantitative detection
*qCHT1-* R	TGCCAAGCGTGTAGGTCATCAAGT	
*qCHT3-* F	CACGAGCCCTACGCCTACAGC	*HtCHT3* quantitative detection
*qCHT3*-R	TGATGTCGGTGCCAGTGAAGGT	
*qCHT4*-F	ACCCCTGGAATGAGTTGTGCC	*HtCHT4* quantitative detection
*qCHT4*-R	TGCGATAGATGGCACTGTTGATG	
*Ht*-*GAPDH*-F	GTCTCCAATGCCTCCTGC	quantitative detection of the reference gene *GAPDH*
*Ht*-*GAPDH*-R	GCACTCCTTGCCTAAGATAACA	

### Chitinase sequence alignment and functional domain analysis

The open reading frame (ORF) sequences and translation protein sequences were predicted by ORF Finder (https://www.ncbi.nlm.nih.gov/orffinder/). The protein sequences were submitted to online analysis software SignalP−6.0 Server (https://services.healthtech.dtu.dk/service.php?SignalP) and SMART (http://smart.embl-heidelberg.de/smart/set_mode.cgi?NORMAL=1) for prediction and analysis of protein signal peptides and domains, respectively. MEGA7 software was used to compare and analyze the coding sequence (CDS) of *H. tientsinensis* and CDS regions of other homologous genes in NCBI GenBank (https://www.ncbi.nlm.nih.gov/). Neighbor-joining (NJ) method was conducted to construct phylogenetic trees and analyze the evolutionary relationships among different species of chitinase in crustacean by MEGA6.0 and FigTree v1.4.4.

### Tissue-specific expression analysis

Quantitative primers were designed based on the full-length cDNA sequences (Table 1), and the expression profiles of *CHITINASE* genes in different tissues (hepatopancreas, gill, muscle, heart, stomach and eye stalk) of *H. tientsinensis* were detected and analyzed by qRT-PCR. The reaction system is as follows: 10 µL 2 × SuperReal PreMix Plus (TIANGEN, China), 2 µL cDNA template (50 times diluted), 0.6 µL forward primer (10 µmol/L), 0.6 µL reverse primer (10 µmol/L), and ddH_2_O to 20 µL. Mixture was put into ABI QuantStudio 3 quantitative PCR instrument (Applied Biosystems, Foster City, CA, USA), and executed the procedure: pre-denaturation at 95 °C for 15 min; denaturation at 95 °C for 10 s, annealing at 55 °C for 20 s, elongation at 72 °C for 20 s, 45 cycles; Dissolution curves were 95 °C for 10 s, 65 °C for 60 s, 97 °C for 1 s. The 2^−ΔCT^ method was used to calculate the relative expression level of target gene relative to internal reference *GAPDH*, and significance analysis was conducted using one way ANOVA by SPSS Statistics 23. The calculation method is as follows: ΔCT = CT^T^-CT^G^; relative transcript level = 2^−ΔCT^ (CT, cycle threshold; CT^T^ is CT value of target gene; CT^G^ is CT value of internal reference gene *GAPDH*).

### *V. parahaemolyticus* challenges in *H. tientsinensis*

*V. parahaemolyticus* strain (ATCC 17802) was bought in China general microbiological culture collection center (CGMCC) and stored at the −80 °C freezer. *V. parahaemolyticus* strain was cultured in the 3%NaCl nutrient agar liquid medium (10 g peptone, 3 g beef extract, 30 g NaCl dissolved in 1 L ddH_2_O, pH = 7.0), and then the bacteria cells were centrifuged at 4,000 rpm for 10 min and suspended by phosphate buffer (PBS) for the further infection experiment.

*H. tientsinensis* were collected in the mudflat and randomly divided into the experiment group (*V. parahaemolyticus* group) and control group (PBS group) after temporary rearing for two days. Each mudflat crab in the experiment group was injected with 50 µL *V. parahaemolyticus* suspension containing 1 ×10^6^ bacteria copies in the second walking leg, while 50 µL PBS was injected into each mudflat crab in the control group. The hepatopancreas of six mudflat crabs were samples at each time point (0 h before injection, and 6 h, 12 h, 24 h, 48 h, 72 h in the post-infection period) in the experiment and control group, respectively. Hepatopancreas was immediately frozen in the liquid nitrogen and stored in the −80 °C freezer for RNA isolation until use, followed by RNA extraction and cDNA libraries construction. *CHITINASE* genes at different time points after injection in hepatopancreas of *H. tientsinensis* were detected and analyzed by qRT-PCR. The relative expression level of target genes at different time points after injection were calculated by the 2^−ΔΔCT^ method as described previously (Livak & Schmittgen, 2001). *GAPDH* was used as the internal reference gene to standardize the results. All primers used were listed in Table 1.

## Result

### *CHITINASE* cDNA cloning

**Figure 1:**
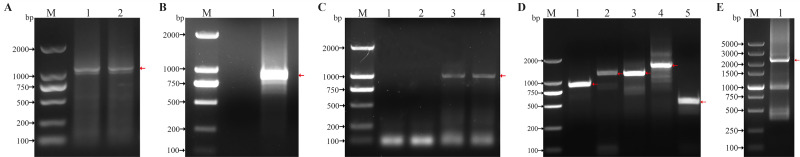
Cloning of conserved region and 5′/3′ cDNA ends of the *HtCHT1, HtCHT3* and *HtCHT4*. (A) Conserved region of the* HtCHT1*. Lane M: DNA marker DL2000 (TAKARA, Japan); Lane 1, 2: conserved region of the *HtCHT1*. (B) Conserved region of the *HtCHT3*. lane M: DNA marker DL2000; Lane1: conserved region of the *HtCHT3*. (C) Conserved region of the *HtCHT4*. lane M: DNA marker DL2000; Lane 1,2: conserved region of the *HtCHT3*; Lane 3,4: conserved region of the *HtCHT4*. (D) 5′/3′ cDNA ends of the *HtCHT1*, *HtCHT3* and 5′ end of the *HtCHT4* cDNA. Lane M: DNA marker DL2000; Lane 1: 5′cDNA ends of the *HtCHT1*; Lane 2: 3′ cDNA ends of the *HtCHT1*; Lane 3: 5′ cDNA ends of the *HtCHT3*; Lane 4: 3′ cDNA ends of the *HtCHT3*; Lane 5: 5′ cDNA ends of the *HtCHT4*. (E) 3′ cDNA ends of the *HtCHT4*. Lane M: DNA marker DL2000; Lane 1: 3′ cDNA ends of the *HtCHT4*.

The primers were designed based on the RNA sequencing results of *H. tientsinensis* and homologous genes of NCBI GenBank, and the conserved regions of *HtCHT1, HtCHT3* and *HtCHT4* genes were cloned. The conserved regions of cDNA were 1,132 bp, 1,027 bp and 980 bp, respectively (Figs. 1A, 1C, Fig. S1), and the 5′/3′ ends of each cDNA were cloned by RACE (Figs. 1D, 1E, Fig. S1).

**Figure 2:**
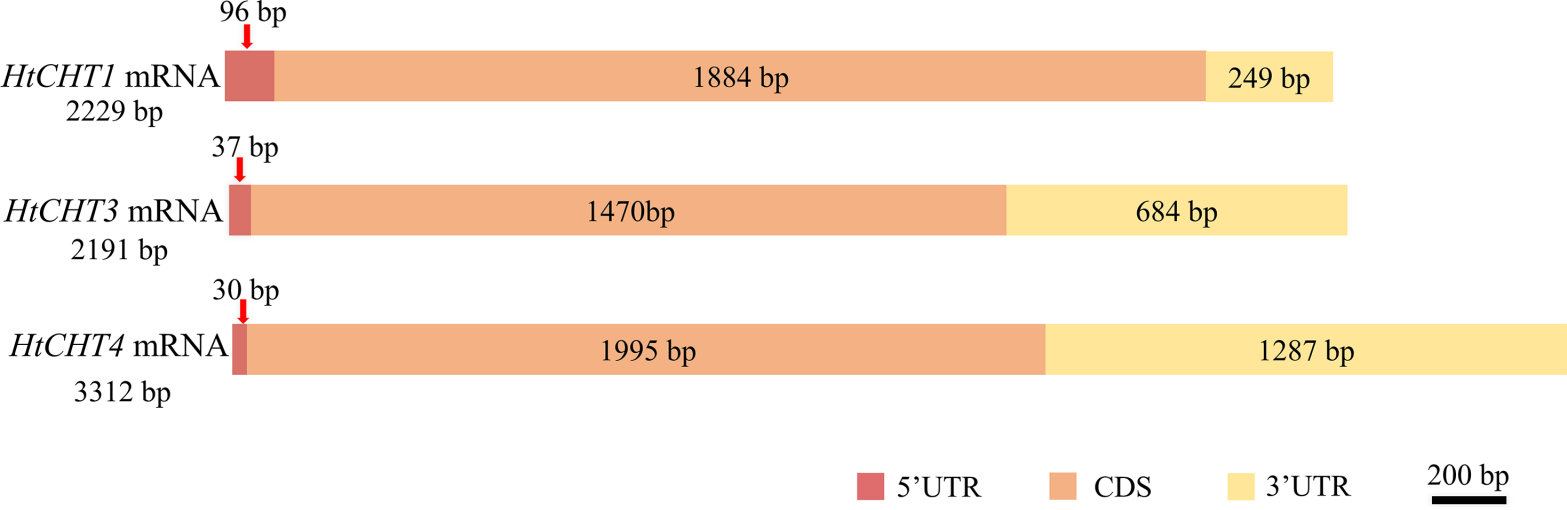
Physical map of the *HtCHT1*, *HtCHT3* and *HtCHT4* genes.

Sequence analysis showed that the full-length cDNA of the *HtCHT1* was 2,229 bp (NCBI ID: OQ025399), including 96 bp 5′UTR, 1,884 bp CDS and 249 bp 3′UTR, encoding 627 amino acids (AA) (Fig. 2). The full-length cDNA of the *HtCHT3* was 2,191 bp (NCBI ID: OQ025400), including 37 bp 5′UTR, 1,470 bp CDS and 684 bp 3′UTR, encoding 684 amino acids (Fig. 2). The full-length cDNA of the *HtC HT4* was 3,312 bp (NCBI ID: OQ025401), including 30 bp 5′UTR, 1,995 bp CDS and 1,287 bp 3′UTR. The code produced 664 amino acids (Fig. 2).

### Bioinformatics and phylogenetic analysis of chitinase

**Figure 3:**
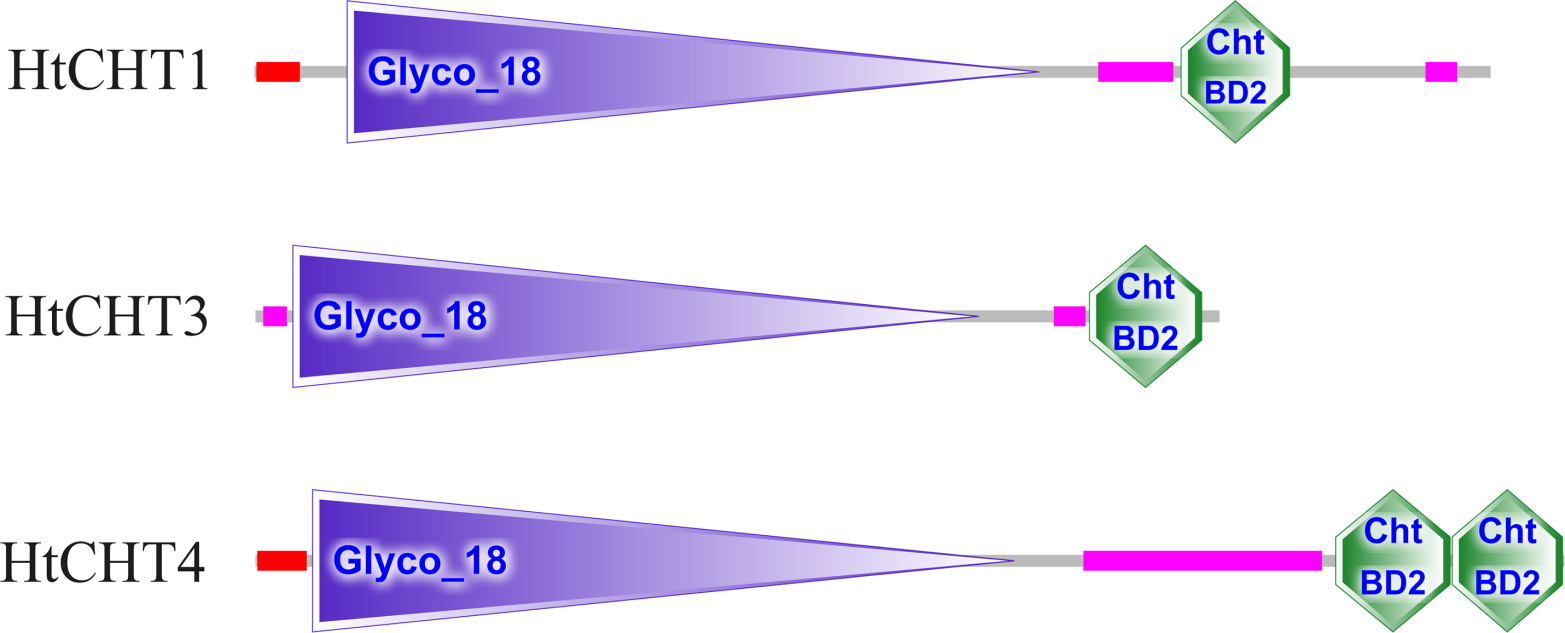
The analysis of functional domains of HtCHT1, HtCHT3 and HtCHT4 protein. The red region represented the signal peptide, the purple triangle writing Glyco 18 in the middle represented the GH18 chitinase family catalytic domain, and the green hexagon writing the ChtBD2 on the right represented the binding domain.

**Figure 4:**
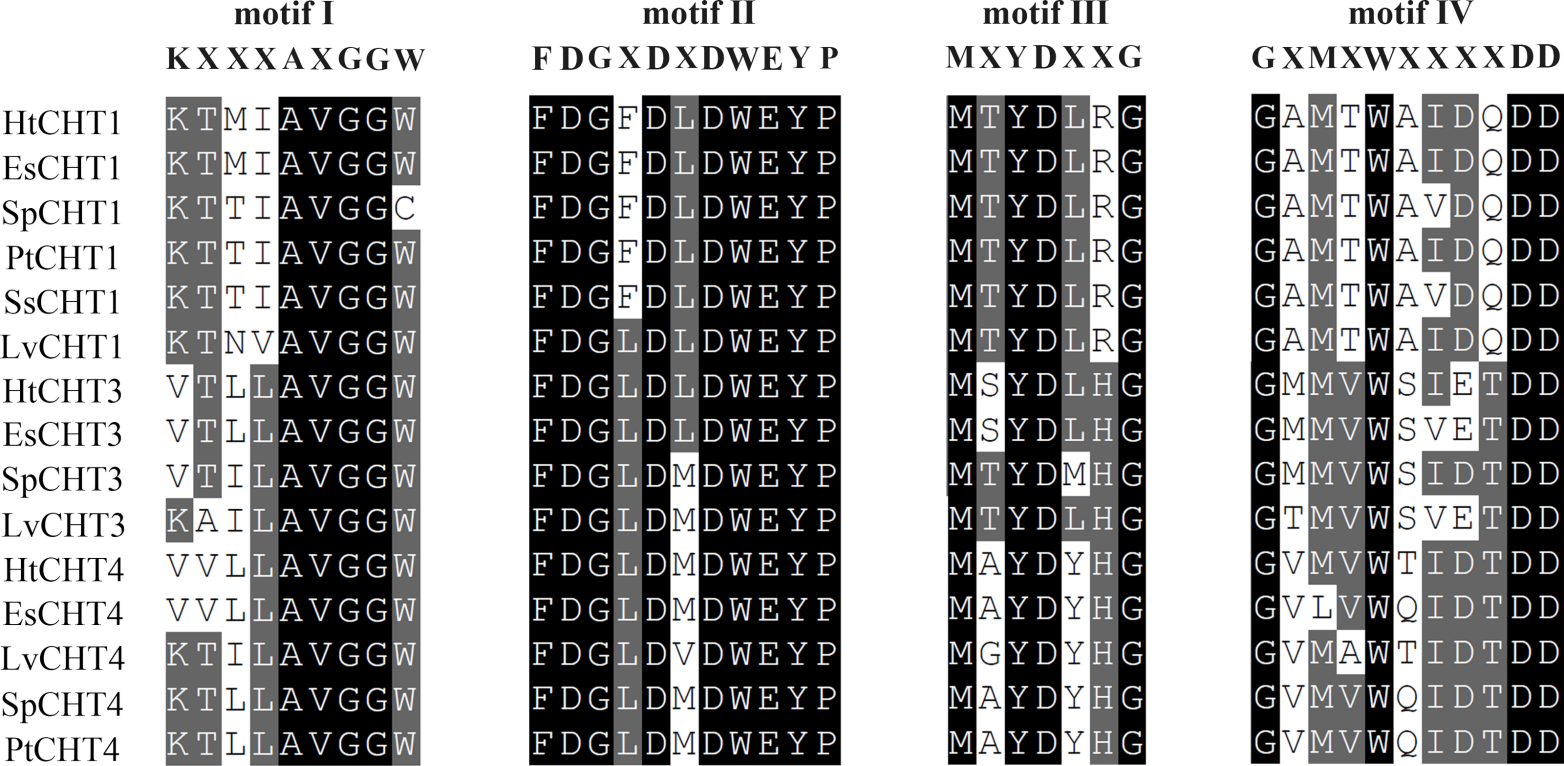
The analysis of the catalytic domains of chitinase family proteins in crustaceans. The prefix Sp, Lv, Es and Pt of chitinases were represented the species of *S. paramamosain*, *L. vannamei*, *E. sinensis* and *P. trituberculatus*, respectively. All of protein sequences used were listed in Table S1.

The analysis of signal peptides and functional domains of the HtCHT1, HtCHT3 and HtCHT4 protein showed that HtCHT1 protein had 23 AA signal peptides at the N-terminal. 47–398 AA was the catalytic domain of glycoside hydrolases 18 family (GH18) of the HtCHT1, and 470–525 AA was the chitin-binding domain (ChtBD2). The HtCHT3 had a GH18 catalytic domain between 19 and 367 AA, and 423–480 AA was chitin-binding domain. The N-terminal of the HtCHT4 had 26 AA signal peptides, while 29–385 AA was also GH18 catalytic domain, and HtCHT4 had two chitin-binding domain (548–606 AA and 607–663 AA) (Fig. 3). There were three highly conserved motif (motif II, motif III, motif IV) in the HtCHT1, HtCHT3 and HtCHT4. However, only HtCHT1 had the complete motif I, and the lysine (K) was replaced by valine (V) between the HtCHT3 and HtCHT4 (Fig. 4).

**Figure 5:**
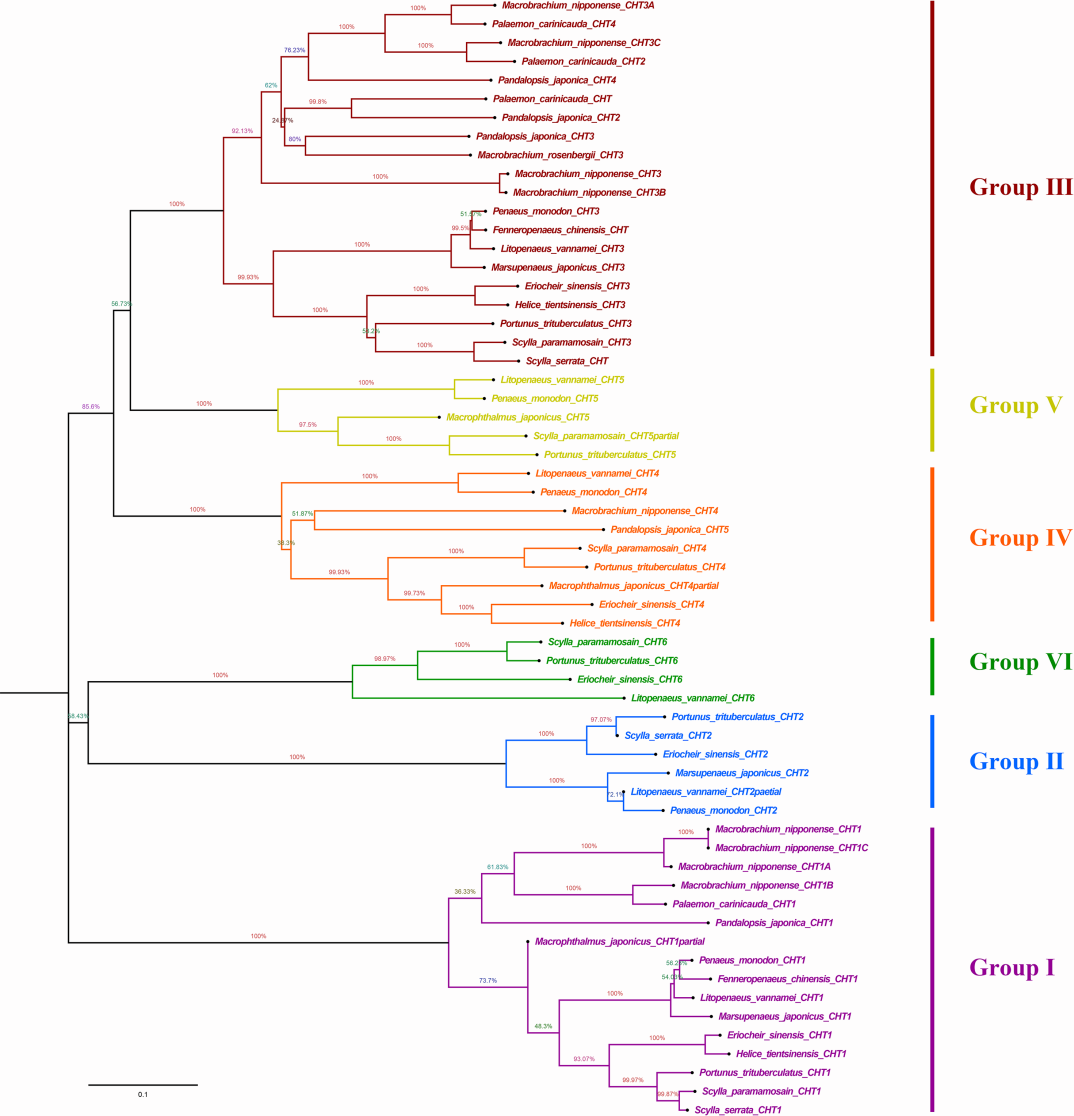
Phylogenic analysis of chitinase protein sequences in crustaceans (neighbour-joining tree). HtCHT1, HtCHT3 and HtCHT4 protein were marker with red five-pointed stars. The NCBI accession numbers of sequences in different crustaceans were listed in the Table S1.

Phylogenetic analysis showed that different chitinases clustered in similar clusters, which were obviously divided into six categories and each kind of chitinases were concentrated in the same category. Crabs and shrimps further aggregated, and the internal genetic distance was relatively closer (Fig. 5).

NJ phylogenetic tree was constructed by alignment the chitinase protein sequences among *H. tientsinensis*, *Eriocheir sinensis*, *Scylla serrata*, *S. paramamosain*, *P. trituberculatus*, *Macrobrachium nipponense, L. vannamei*, *Fenneropenaeus chinensis*, *Penaeus monodon*, *P. japonica*, *Marsupenaeus japonicus* and *Exopalaemon carinicauda.* Phylogenetic analysis showed that HtCHT1, HtCHT3, and HtCHT4 of *H. tientsinensis* were all in the corresponding cluster, Group I, Group II, Group IV, respectively. The EsCHT1, EsCHT3 and EsCHT4 of *E. sinensis* (Varunidae) had the closest genetic distance with HtCHT1, HtCHT3, and HtCHT4, while *M. nipponense* (Palaemonidae) had the farthest genetic relationship (Fig. 5). Comparing the HtCHT1, HtCHT3, HtCHT4 with the EsCHT1, EsCHT3, EsCHT4, the conservations of protein sequences were 79.1%, 92.9% and 65.5%, respectively.

### Tissue-specific expression analysis

**Figure 6:**
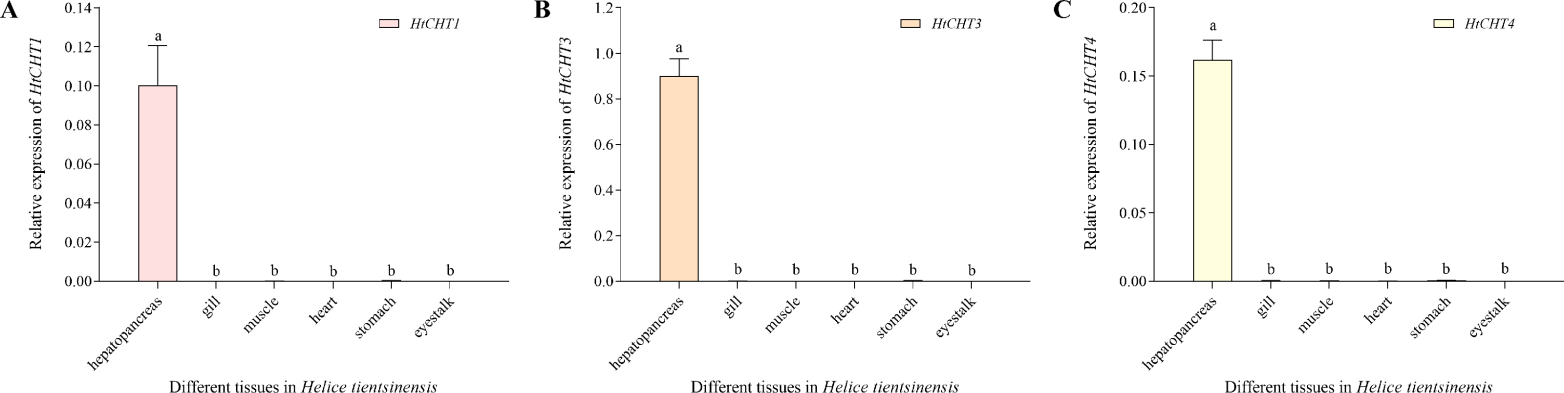
Expression profiles of the *HtCHT1, HtCHT3* and *HtCHT4* in *H. tientsinensis*. qRT-PCR was used to examine the expression of the *HtCHT1* (A), *HtCHT3* (B) and *HtCHT4* (C) in the hepatopancreas, gill, muscle, heart, stomach and eye stalk samples from *H. tientsinensis*, with *GAPDH* as a reference. The data are the mean ± SD of three independent biological replicates. Lowercase letters indicate statistical significance at *P* < 0.01 by Duncan’s test.

The expression of the *HtCHT1, HtCHT3* and *HtCHT4* of different tissues/organs (hepatopancreas, gill, muscle, heart, stomach and eye stalk) in *H. tientsinensis* was analyzed by qRT-PCR. The expression of the *HtCHT1, HtCHT3* and *HtCHT4* showed the tissue-specific expression and similar expression profiles. They specifically expressed in the hepatopancreas, and the expression levels of three genes were significantly higher than other tissues and organs (*P* < 0.01) (Fig. 6).

### Expression analysis of *CHITINASES* in *H. tientsinensis* after bacterial infection

**Figure 7:**
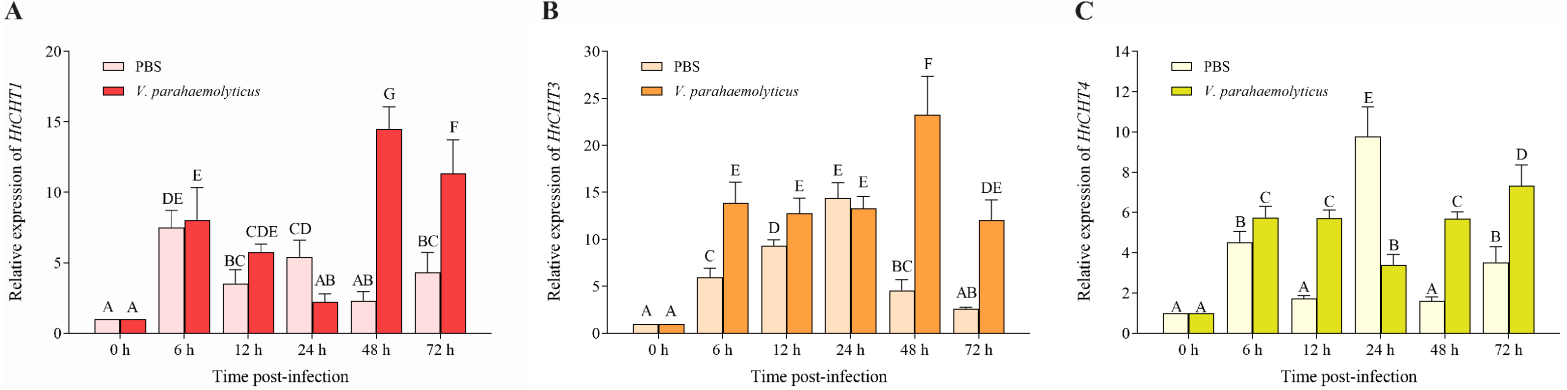
Relative expression levels of the *HtCHT1, HtCHT3* and *HtCHT4* in the hepatopancreas of *V. parahaemolyticus*-infected *H. tientsinensis*. qRT-PCR was used to examine the expression of the *HtCHT1* (A), *HtCHT3* (B) and *HtCHT4* (C) in the hepatopancreas of *H. tientsinensis* before (0 h) and after (6 h, 12 h, 24 h, 48 h and 72 h) *V. parahaemolyticus* infection. *GAPDH* was used as the reference gene. The data are the mean ± SD of three independent biological replicates. Capital letters indicate statistical significance at *P* < 0.05 by Duncan’s test.

To study the biological functions of three *CHITINASES*, the expression of the *HtCHT1, HtCHT3* and *HtCHT4* in hepatopancreas was detected at 6 h, 12 h, 24 h, 48 h and 72 h after *V. parahaemolyticus* infection (Fig. 7). *HtCHT1* in hepatopancreas infected with the *V. parahaemolyticus* showed significant up-regulation expression by more than 14-fold at 48 h, compared to that in the PBS group, with a tendency to decrease at 72 h, showing up-regulation (11.3-fold), compared to the PBS group (*P* < 0.05) (Fig. 7A). Significant up-regulation of *HtCHT3* was observed from the 48 h (23.2-fold) to 72 h (12.0-fold) after infected with the *V. parahaemolyticus* as compared with PBS solution (*P* < 0.05) (Fig. 7B). The expression of *HtCHT4* was significantly up-regulated at 48 h (5.6-fold) and 72 h (7.3-fold) and kept a significantly up-regulated pattern until the tested 72 h compared to the PBS group in hepatopancreas (*P* < 0.05) (Fig. 7C).

## Discussion

Chitinase plays an essential role in crustaceans, including the degradation of exoskeletons involved in the molting growth of crustaceans (Huang et al., 2010; Proespraiwong, Tassanakajon & Rimphanitchayakit, 2010), digestion of chitin food in the peritrophic membrane of the digestive tract (Salma et al., 2012; Zhang et al., 2014) and serving as immune genes involved in pathogenic immunity (Tan, Degnan & Lehnert, 2000; Zhou et al., 2018). As an important ecological species with high economic value, *H. tientsinensis* is widely distributed throughout the mudflats of the East Sea, the Yellow Sea and the Bohai Sea in China (Wang et al., 2019). However, chitinase related studies has not been reported in *H. tientsinensis.*

In this study, the full-length of the *HtCHT1*, *HtCHT3* and *HtCHT4* cDNA were successfully cloned and analyzed from *H. tientsinensis* by conservative sequence amplification combined with RACE technique, and then structural and functional domain analysis, phylogenetic analysis and expression profile analysis were carried out.

Structural and functional domain analysis showed that all three chitinase proteins had different signal peptide sequences, GH18 catalytic domains and chitin-binding domains. Their CDS sequences information were highly similar to other shrimps and crabs of crustacean species such as *E. carinicauda* (Duan et al., 2014), *E. sinensis* (Li et al., 2015), *M. nipponense* (Zhang et al., 2014), *P. trituberculatus* (Song et al., 2020), *L. vannamei* (Huang et al., 2010) and *P. monodon* (Proespraiwong, Tassanakajon & Rimphanitchayakit, 2010). The results showed that three chitinase proteins in *H. tientsinensis* all belonged to the GH18 chitinase family, which had four highly conserved motifs (Fig. 3). HtCHT1 and HtCHT4 proteins contained the signal peptide, indicating that they were secretory proteins. But, HtCHT3 lacked the significant signal peptide, which is consistent with the study in *E. sinensis* (Li et al., 2015) and *L. vannamei* (about 10 AA) (Huang et al., 2010). Besides, the HtCHT4 contained two chitin-binding domains, which was consistent with the studies in *L. vannamei* (LvCHT4) (Huang et al., 2010)*, P. monodon* (PmCHT4) (Zhou et al., 2017) and *S. paramamosain* (SpCHT4) (Zhou et al., 2018). It suggested that HtCHT4 could preferably combine the chitin and decompose insoluble chitin, and it would enhance the efficiency of chitin degradation. HtCHT1, HtCHT3 and HtCHT4 had four highly conserved motifs (motif I–IV) in GH18 catalytic domain which suggested that chitinases were conserved in terms of evolution. Although the HtCHT3 and HtCHT4 had an amino acid difference (K →V) with the common sequence of motif I (KXXXAXGGW), we speculated it didn’t affect the functions of the HtCHT3 and HtCHT4, because the sequences of the EsCHT3 and EsCHT4 in *E. sinensis* as well as the SpCHT3 in *S. paramamosain* had the same amino difference.

Zhang et al. (2017) divided chitinase sequences into seven groups in crustaceans (*E. sinensis*, *S. serrata*, *S. paramamosain*, *P. trituberculatus*, *M. nipponense, L. vannamei*, *P. japonica*), such as the I, II, III, IV, V, VI and VII groups base on multiple sequence alignment and NJ phylogenetic tree construction (Zhang et al., 2017). According to multiple sequence alignment and NJ phylogenetic tree construction, different species of chitinase proteins were clustered in one group, while *H. tientsinensis* and *E. sinensis* had the highest chitinase homology and clustered in the same branch, followed by other crabs. They had lower homology with shrimp families, and the farthest relationship with *M. rosenbergii* in family Palaemonidae (Fig. 4). Both *H. tientsinensis* and *E. sinensis* belonged to the family Varunidae, and HtCHT1, HtCHT3, HtCHT4 and EsCHT1, EsCHT3, EsCHT4s had the relatively higher homology. In addition, they were located in a closer branch in the phylogenetic tree. It suggested that more closely related species had higher sequence homology.

*CHITINASE* genes belonged to a multiple gene family. Different kinds of *CHITINASE* genes are expressed differently in different tissues/organs of crustaceans and insects (Ye et al., 2019). The expression of the *HtCHT1*, *HtCHT3* and *HtCHT4* in different tissues of *H. tientsinensis* was analyzed by qRT-PCR. The results showed that the expression of the *HtCHT1*, *HtCHT3* and *HtCHT4* was tissue-specific and was specifically expressed in hepatopancreas, which was similar to the result of the *SpCHT1*, *SpCHT3* and *SpCHT4* in *S. paramamosain* (Zhou et al., 2018) and the *LvCHT1*, *LvCHT3*, *LvCHT4* in *L. vannamei* (Huang et al., 2010).

Hepatopancreas is a vital secretory organ of digestive enzymes and producing immune factors in crustaceans (Cao et al., 2021). Therefore, it is speculated that *HtCHT1*, *HtCHT3* and *HtCHT4* may play an important role not only in the digestion but also in the immune of *H. tientsinensis*. In order to further explore the biological functions of the *HtCHT1*, *HtCHT3* and *HtCHT4*, the expression level of three genes in hepatopancreas were analyzed after *V. parahaemolyticus* injection at different time points (0 h, 6 h, 12 h, 24 h, 48 h and 72 h). Compared to the expression level in the PBS group, all three genes in the *V. parahaemolyticus* group were significantly improved at 48 h and 72 h time points after injection. Additionally, *HtCHT1* and *HtCHT3* reached the peak at 48 h post injection, and decreased at 72 h, whereas *PtCht-1* in *P. trituberculatus* showed a similar expression pattern (Song et al., 2020). Meanwhile, *HtCHT4* expression was obviously up-regulated after 48 h, but peaked at 72 h after injection. Moreover, the expression level of *PmChi-4* in *P. monodon* was also increased after the infection with *V. harveyi* and *Streptococcus agalactiae* (Zhou et al., 2017). Results suggested that the *HtCHT1*, *HtCHT3* and *HtCHT4* were involved in the immune defense of *H. tientsinensis*.

## Conclusions

In conclusion, we obtained the complete mRNA sequences of the *HtCHT1*, *HtCHT3* and *HtCHT4* from *H. tientsinensis*. Their predicted HtCHT1, HtCHT3 and HtCHT4 proteins belonged to the GH18 chitinase family and had four highly conserved motifs as well as GH18 catalytic domains and chitin-binding domains. Phylogenetic tree analysis showed that the HtCHT1, HtCHT3 and HtCHT4 proteins belonged to Group I, Group III and Group IV, respectively, and had the closest genetic relationship with *E. sinensis*. The *HtCHT1*, *HtCHT3* and *HtCHT4* were specifically expressed in hepatopancreas, and significantly up-regulated after *V. parahaemolyticus* injection over 48 h compared with that of PBS injection, which speculated that these three genes might play an important role in the immunity and digestion of *H. tientsinensis*. This study will provide new insight into the function of different genes in the *CHITINASE* family.

##  Supplemental Information

10.7717/peerj.15045/supp-1 Supplemental Information 1 Full-length of the *HtCHT1*, *HtCHT3*, *HtCHT4* cDNA and the corresponding proteins The CDS region of each gene was marked with green background.

10.7717/peerj.15045/supp-2 Supplemental Information 2 Raw data

10.7717/peerj.15045/supp-3 Supplemental Information 3 Raw data

10.7717/peerj.15045/supp-4 Supplemental Information 4 Uncropped gels of Fig. 1

10.7717/peerj.15045/supp-5 Supplemental Information 5 Accession numbers of NCBI Genbank used in phylogenetic analysis

## Additional Information and Declarations

### DNA Deposition

The following information was supplied regarding the deposition of DNA sequences: The sequences of HtCHT1, HtCHT3 and HtCHT4 are available at GenBank: OQ025399, to OQ025401, and available in the Supplementary File.

### Data Availability

The following information was supplied regarding data availability: The sequences of gene and protein are available in the Supplementary File.
