# Supplementary material for: Cloning, characterization, and expression analysis of the CHITINASE gene family in Helice tientsinensis
Source: PeerJ. 2023 Mar 14;11:e15045. doi: 10.7717/peerj.15045 (PMC10022498; doi:10.7717/peerj.15045)
Supplement: Supplemental Information 1 — The CDS region of each gene was marked with green background. [file peerj-11-15045-s001.docx]

**Supplemental data S1：**

**Full-length of the *HtCHT1*, *HtCHT3*, *HtCHT4* cDNA and the corresponding proteins.**

The CDS region of each gene was marked with green background.

*>HtCHT1*_cDNA

acatggggacacagtccattattaattactgtacgtgtgtcgtcttcgcgtcgaaccctcaacacttaccccggtcgattgtgtagcgatctcattatgaatataaagggagcggtgtgttgcgggacgctggtggcgctgctgctggcgacctgcgtcagcgccgagcctcgctacgctaggccgggagaccacgagccaatgccgcacccgcgcgcgacgcctgaccgcctggcgcgccgcgtgtgttactacgagacgtgggccaagtaccggcccgaagaggtgcactacgacatcgaggacatccccggggatatgtgcacccacctcatctacaccttctgcggcgtgtccaacgtgacatgggaggtcctcatgttggacccggagctcgatatcaacgccgatggttaccggcgcttcgttgccctgaaggagaagtaccccgacgtgaagacaatgatagccgtgggaggctgggcggagggcggaaagaagtactcccagatggtgagcgtcaaggagcgccgagacactttcatcaggagtgttgtccagttgctgacggactacggtttcgacggcttcgaccttgattgggagtaccccggggcgactgaccgcggcggcacctacgctgacaaagaaaactacctgctgctggtccaggaacttcgcgaggccttcgacgccgtgggtatgggctgggacctgaccgccgccgtgcccgtcgccaggttcagactgcaggagggctaccacgttcccgagctgtgcagcctactggatgccatacacttgatgacctacgacctgcgcgggaactggtgcgggttcgcggatgtccactctatgctgtacatgcggcctggcctcgacgagtggtcctacgagaagctgaacgacaatgacggtatgctgctgtgggaggagttcgggtgcccgcgcgacaagatggtccttggcacgcccttctacgcccgcacctacacgcttggcagccccgacaacaacgacctgcacgctcccatcaagaagtgggagggcggcggcttgcccggcccctacaccaacgccaccggcaccctcgcctacttcgaggtttgcaagatgatgctggacgactctgcctgggtggatcgctacgacgacgtgggactcgttcccttcacgcacaaagatgaccagtgggtgggctatgaggatcccgacagcctccagatcaagatggactacataaaggagcaggggctgttgggggccatgacgtgggctattgaccaagacgactacctgggctggtgcggccgcggcgtcaaccccatgatgacggttctctacgaggggatgaaggactacatagtgcccgtggccccaaccgtcacctccaccaccacgagcccgtggtggacgcagccttccaccaccaccaccaccagggaccccaacaagtccaccaccacgcgggaccccaaccaacctaccaccactctgggccccctagactgcaacctggccgcatactggccccacgaggactgtgacaagtattactggtgctacggtggcgtgccgcacctggagcagtgtcccgacggcacagtgtggaacaatgccggggcgtactgcgactggccggagaacgttgacacctcgcactgcaacatgcccgcgttcgcgcttcaacccaaacagcacgatggaccccctcccaagaccaagcacatcaagcccaaagctccagtcaaacccaaggaagttcatcccgccccgccgcccaaacatgaggaaatccccaaggctcaggccaagcccctggcccatgctaaactcgccgcaaacaaaccttcacccaagcttaacgaggtcaaccattctcaccccaagagtaaaagtgcctctcaccccaaagctgctcccaaactcgagaacgtcaaaatcccccctctaatgaaggtactgaataggctaccctaagcgccacgcatatcccaggcgacaagataatggcctattatggggtcattcaaagtactgtacataactacaaactattctcaatgttttgtttcttgacctaaatatagttcatatctacttgaatatccagaacggtgctctcaatttaagtatttgaatgttacgactcaaataatatttaaccagcaaagtaccaataaataaaagttttgttagcaaaaaaaaaaaaaaaaaaaaaaaaaaaaa

>HtCHT1_protein

MNIKGAVCCGTLVALLLATCVSAEPRYARPGDHEPMPHPRATPDRLARRVCYYETWAKYRPEEVHYDIEDIPGDMCTHLIYTFCGVSNVTWEVLMLDPELDINADGYRRFVALKEKYPDVKTMIAVGGWAEGGKKYSQMVSVKERRDTFIRSVVQLLTDYGFDGFDLDWEYPGATDRGGTYADKENYLLLVQELREAFDAVGMGWDLTAAVPVARFRLQEGYHVPELCSLLDAIHLMTYDLRGNWCGFADVHSMLYMRPGLDEWSYEKLNDNDGMLLWEEFGCPRDKMVLGTPFYARTYTLGSPDNNDLHAPIKKWEGGGLPGPYTNATGTLAYFEVCKMMLDDSAWVDRYDDVGLVPFTHKDDQWVGYEDPDSLQIKMDYIKEQGLLGAMTWAIDQDDYLGWCGRGVNPMMTVLYEGMKDYIVPVAPTVTSTTTSPWWTQPSTTTTTRDPNKSTTTRDPNQPTTTLGPLDCNLAAYWPHEDCDKYYWCYGGVPHLEQCPDGTVWNNAGAYCDWPENVDTSHCNMPAFALQPKQHDGPPPKTKHIKPKAPVKPKEVHPAPPPKHEEIPKAQAKPLAHAKLAANKPSPKLNEVNHSHPKSKSASHPKAAPKLENVKIPPLMKVLNRLP*

*>HtCHT3*_cDNA

acatggggagagctcccagcaccgctcatccaacaccatgaagactctaccgcttctcctcgccgccctggcctccctcgccctcacagagggcgtgatggtgtgctactacgtgtcgtgggccgtgtaccgacagggcgacgggaagttcgacgtggaggacatcgacccgggcatctgtactcacctgatctttggctttgctggtctgggcttcgacaacaagatcagggtgctggacccttggaacgagctctgcgacaattacgggaagtgtgccttcgatcgcttcacggcgctcaagaagaagaacacgaacttggtgaccctgttggctgttggcggctggaacgagggctctgcgaagtactccagtatggcatcgagtgcggccaccagaaagatcttcgtagactcctccatcgaactactcaaggaacacgacttcgatggtctggatctcgactgggagtacccgacccagcgtggaggctcccccgaggacaaggccaacttcattctcctcctgcaagacctgaaggacgctctgcacgccaacgggatgatgctgagcgctgctgtgtccgccggcaaggctaccatcgacgaggcttatgacatccccggcgtggcccagaacctcgacctcatgaacctgatgtcctatgacctgcacggcgcctgggactcctacacgcaccaccagtccggcctctacgcccaccccgatgacactggcgacaacgtgtatctgaaccaagacttcgccatcaactactggatcgacggtggaatgcctgccgagaagatcgctcaaggtgttcctctctacgggcgatgctggacactgagcagcgccgacaataccggttactacgcccccgccacccaacccggcatggcaggaccctacaccagaagccccggattcctcggctacaatgagatctgtgaggccattgagacccgcggctggacggttgtgaacgaccccgccatgcacgagccctacgcctacagcatcgaggacaaccgtatttggtgctcctacgatgacgaggcctccgtgacgaccaaggctctgtacgccaaggatcacggactggccggcatgatggtctggagtatcgagaccgacgatttcttgggcaagtgtggccgtgagttcaacctgatcaagaccatggtggagaccttcacgggcaccgacatcacgccgcccccgacccagtccacgaccaccagggaccccagcgagaccactccatcccccgtcaccaagcccaccacgccaccccccgacggcgtgtgcaataggcctggcatcaacgctgaccccgacaactgccatcactactggctttgcgcccagaacaccaacggcggctacgatgcgacggaagagccctgcgctgacggaactctcttcaaccccgagtccttcatctgcgactgggattacgtcgtgtgcgctctccccgacacgtgcgttaacgactgcgcctaaacgacacattacacacacacactaacacacacgtacatgctttcatacacatagagacacacatacacggacggacattcacgcacacacatacacataagctttcccctcaggctgaacggagacgcacacggacacgtacacaacaccaccaccaccaccaccaccaccataactataccccaaaacacttaagtctcccttgtttccatattccacggcttaaacaaattcaaatcaaggacaaactgaaataagagaaagaaaacgaagtgatggtgatcttagtggaggtgatggtgatagagtatagtggtgattatggtggtggtggttgtggtgatggttaggaagagttacagcaagaaagaaatattaagcattgaaaaagtacaaatataaagaaagttaaaaagtgtcttagatgagtaacataaaatacatggaagtcgcgggggggaatagaaagcattgaagataagtacaaaaaaataaagaaggttaaaagtgtcttagagagttaataaaggagaaatagaggttaaaactatatagaagcgggagaggcgagacaataattagtcatgtacggaaaaaaatgtcaggataaattcaaatacgtgacattttttttgttccttcaataaatggctaaaaacataaaaaaaaaaaaaagaaaaaaaa

*>*HtCHT3_protein

MKTLPLLLAALASLALTEGVMVCYYVSWAVYRQGDGKFDVEDIDPGICTHLIFGFAGLGFDNKIRVLDPWNELCDNYGKCAFDRFTALKKKNTNLVTLLAVGGWNEGSAKYSSMASSAATRKIFVDSSIELLKEHDFDGLDLDWEYPTQRGGSPEDKANFILLLQDLKDALHANGMMLSAAVSAGKATIDEAYDIPGVAQNLDLMNLMSYDLHGAWDSYTHHQSGLYAHPDDTGDNVYLNQDFAINYWIDGGMPAEKIAQGVPLYGRCWTLSSADNTGYYAPATQPGMAGPYTRSPGFLGYNEICEAIETRGWTVVNDPAMHEPYAYSIEDNRIWCSYDDEASVTTKALYAKDHGLAGMMVWSIETDDFLGKCGREFNLIKTMVETFTGTDITPPPTQSTTTRDPSETTPSPVTKPTTPPPDGVCNRPGINADPDNCHHYWLCAQNTNGGYDATEEPCADGTLFNPESFICDWDYVVCALPDTCVNDCA*

*>HtCHT4*_cDNA

acatggggatacttcacgaagctctgcctcatgtatcgacacaaactcttgctctctctggcggtgggactcctggcagccctcacgctgccttcccccgcccaagcctacggagatgacgtggtgtgttacttctcgtcgtgggcgcggtggcggcctggcaacggcatgttcgacgtggaaaacatcgaccccttcctctgcacgcacgccatcttcagcttcgcgggcctcagcaacgtcacgtgggagctcgaggtgctggacccctggaatgagttgtgccccagcgaagagggcggctactactgcgcctatgaccgattcacagcgctgaaggagatcaacccagacctggtggtgctgctggcggtgggcggctggcgggagggctccgaggactactccgtgatggcagcagacccagccaaaagaaagaccttcatcaacagtgccatctatcgcatgtacaaacacggcttcgacggcctggacatggactgggagtaccccaccgaccgcggcggctcccccgaggacagggccaacttcgtgctgctgatgcaggagttccgggcggcctttgacaagatgaccaatccgctcatgctgaccttcgccgcggcagccggcaaggacgtaatcgaccaggcgtacgacgtgtcgcagctggtgcccctggtggacaagtggcacgtgatggcctacgactaccacggcgcctgggagaacttcacgcaccaccacgcgaccctctgcggctactatctcgacccggaggagttccagaccttcaacgtgaagttcacagccgagtactacctgagcctcggcgtgcccaaggaaaagatggtgataggcatcgccacctacggccgctgctggacgctggagagcctcgacaacacggggatatacgccaccgctattggcccagggcctgctgggccctacatacagatcccggggacgctgggcttcaatgagatctgcgagaggcttctggctgccggcgacgacgactgcgtggtggtgcatgacccgaacctcctggagccgtacttctactgccacagcgacaagatctggtgcagctacgacgacggggactctgtctacttgaaggctcgttacgcccgcaacctcggcctggccggcgtgatggtgtggacaatcgacacggatgacttccagccgttctgctactcggagccgttccacctcgttcacgagatgaagcgtgcgctcgaggagccggcgggcggagacgaattggtgtgtcaggctacacctacagacacaactacagattcaactacagacacaactacagattcaactacagacacaactacagaatcaactacagacacaactacagaaacaactacagaaacaactacagaaacaactacagaaacaactacagaaacaactactgacacaactacagacacaactacagacacacctataaaaacaactacagaaacaagtactgacacaactacagaatcaactacagacacaactacagaaacaactacagaaactacaagcacaacgactgacacatctagacacaccacattaacagacactagcacctatacagtccctgacaccacaacaatctccatctccaccaccacccgccacccgagtatccgccctgactgcacggggctcgctgatggcaccaccttcccacacagcgactgcaacaagtactgggagtgtgtcaacgaggtcggcatcctggagctctgcagccctggcactgtctgggatgatgagcttaagatctgtaactgggaggaccaagtggacacctcgggctgctacacgtgggcctgcagcgtggacaacacctactacccccaccctgactgcgacaagtacttctggtgctaccaaggctctccacatcaggagcagtgtcctgacggtaattactggagccaagaactgacgcagtgtgtgaggccctcggacgccgacacctccaagtgcaacatcccctgatgaggttggccccgtgtctcctgcctgtgtagcattaggactcactgcaccagcacatggtgggcattaaggttaaggcactgtttggagtaaatcaatctgtcaagaaaaaaatcaagctagatctaaattaagttgtaaacagcacttttagaggcaataactattagtacatcatattcaatttgtagtgtagtggtactaaagaaaggtctgctgtctgtattagcaaggctgtgtgagcaactgttgtgtctcaaaatgcactgttaaggaaggagtgggctctgaaatctgtttcatttacgcacactaaatctaacaaaaataacaaattgacagaagtttgaaagtggaatttttaacaacttgattgaattgtttgtaaaaatagtaacaaaattttgttgtgtactcaacccttaatactgccaaaatatataaggttgtgtcttgaagagtcatatataatctgttattgttaatgtttttcctagtacagtaatacacagcatgaatagtttgcagcctctgccctgagactatcaaagtttgcattatttggctagtagtttgcatggaagacacttctctttgtagcaaagcacaaagcactggaaatgttatgttgggtcagcggagtgagtggcaaaaagggtgaaggtggagggcataggagtgcagagtcctgcagctgcagggcatcagcctgtagcagtgacttaactgctgctcctttgccctccctgcccactgcctggtgttgagagggattgttttcttgttttcactggaaggagcaggttggattatactggaggtgctggctgttgtaccttgtgtgccagcccctacagctgtaaacaagtggtgtagcagccagaacttattccagttagggaacttcatccagtgtgaggctgtaacatgtcccttctggatgatgaaaaacaaagcttctgaggccaagatatatgcttactcagtcgttttacccttaatgactttgaatatgaacacctaggtgaatcacaacctcaagaataaaaggacattgtatggctggtgttaacagtaataatgcatgacaggcttctcataagaatgatgtttttccttctaagtagttttagactatttacagtctcttcatgtaaaagcaatacttatattttgtattgcaagaccaaatattagtgactgtacataagaaacatttagttacttagtacttatataaactaaccaaatgctaaaagaaaaaaaaaaaaaaaaaaaaaaaaaaaa

*>*HtCHT4_protein

MYRHKLLLSLAVGLLAALTLPSPAQAYGDDVVCYFPSWARWRPGNGMFDVENIDPFLCTHAIFSFAGLSNVTWELEVLDPWNELCPSEEGGYYCAYDRFTALKEINPDLVVLLAVGGWREGSEDYSVMAADPAKRKTFINSAIYRMYKHGFDGLDMDWEYPTDRGGSPEDRANFVLLMQEFRAAFDKMTNPLMLTFAAAAGKDVIDQAYDVSQLVPLVDKWHVMAYDYHGAWENFTHHHATLCGYYLDPEEFQTFNVKFTAEYYLSLGVPKEKMVIGIATYGRCWTLESLDNTGIYATAIGPGPAGPYIQIPGTLGFNEICERLLAAGDDDCVVVHDPNLLEPYFYCHSDKIWCSYDDGDSVYLKARYARNLGLAGVMVWTIDTDDFQPFCYSEPFHLVHEMKRALEEPAGGDELVCQATPTDTTTDSTTDTTTDSTTDTTTESTTDTTTETTTETTTETTTETTTETTTDTTTDTTTDTPIKTTTETSTDTTTESTTDTTTETTTETTSTTTDTSRHTTLTDTSTYTVPDTTTISISTTTRHPSIRPDCTGLADGTTFPHSDCNKYWECVNEVGILELCSPGTVWDDELKICNWEDQVDTSGCYTWACSVDNTYYPHPDCDKYFWCYQGSPHQEQCPDGNYWSQELTQCVRPSDADTSKCNIP*
