# Supplementary figures and images for: Cloning, characterization, and expression analysis of the CHITINASE gene family in Helice tientsinensis
Source: PeerJ. 2023 Mar 14;11:e15045. doi: 10.7717/peerj.15045 (PMC10022498; doi:10.7717/peerj.15045)

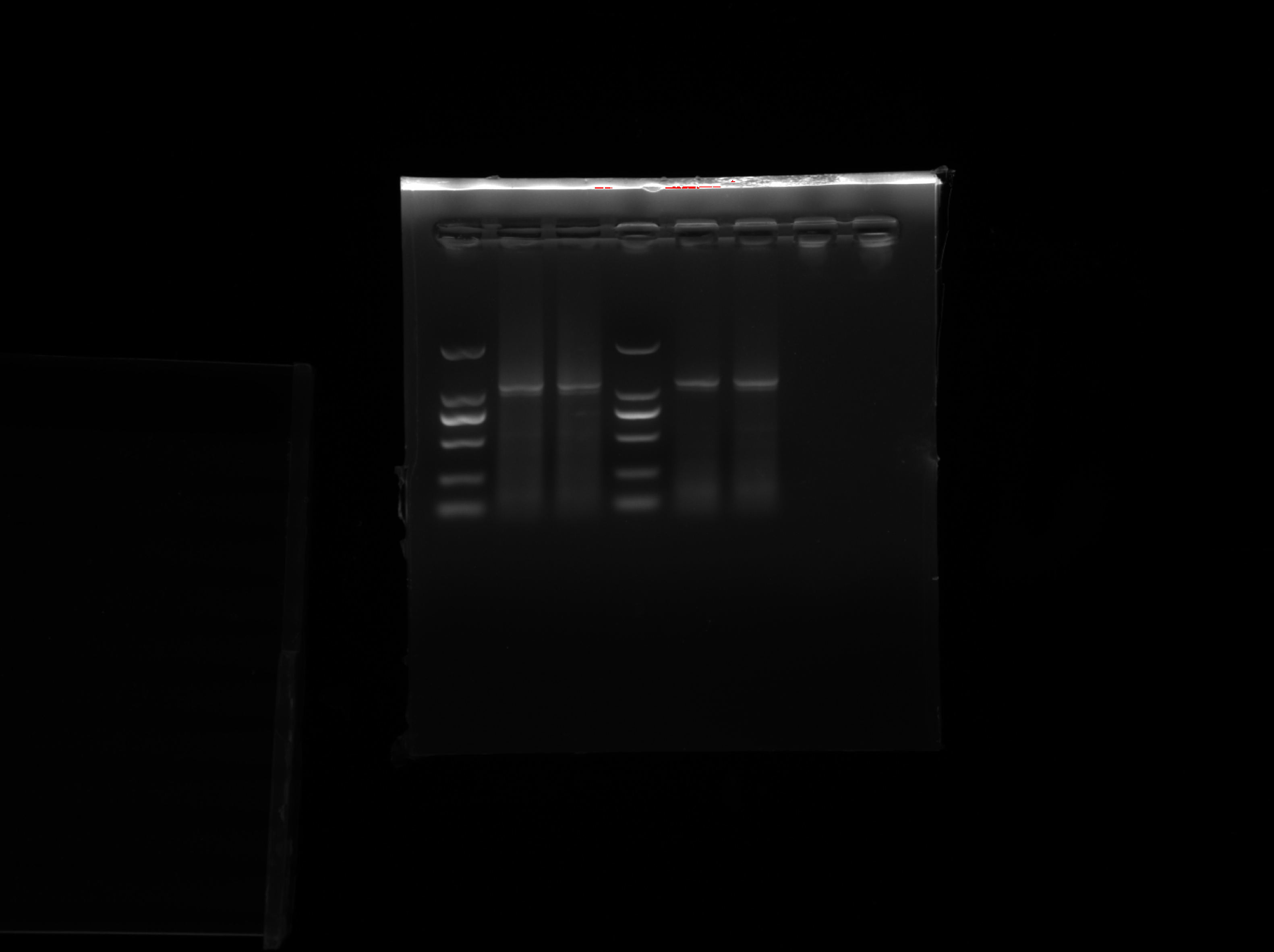

Supplement: Supplemental Information 4 [file peerj-11-15045-s004.zip › Figure 1A.png]

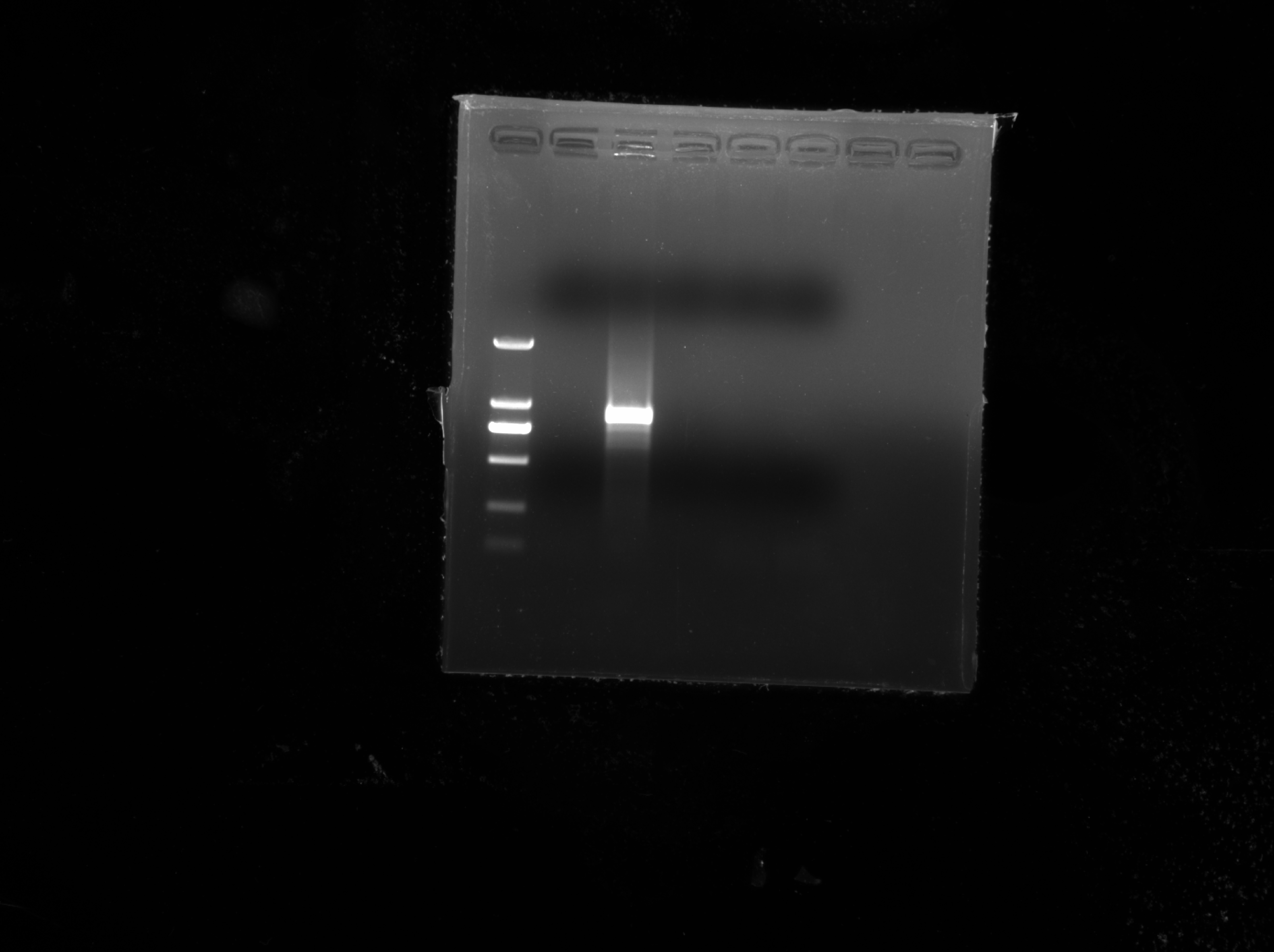

Supplement: Supplemental Information 4 [file peerj-11-15045-s004.zip › Figure 1B.png]

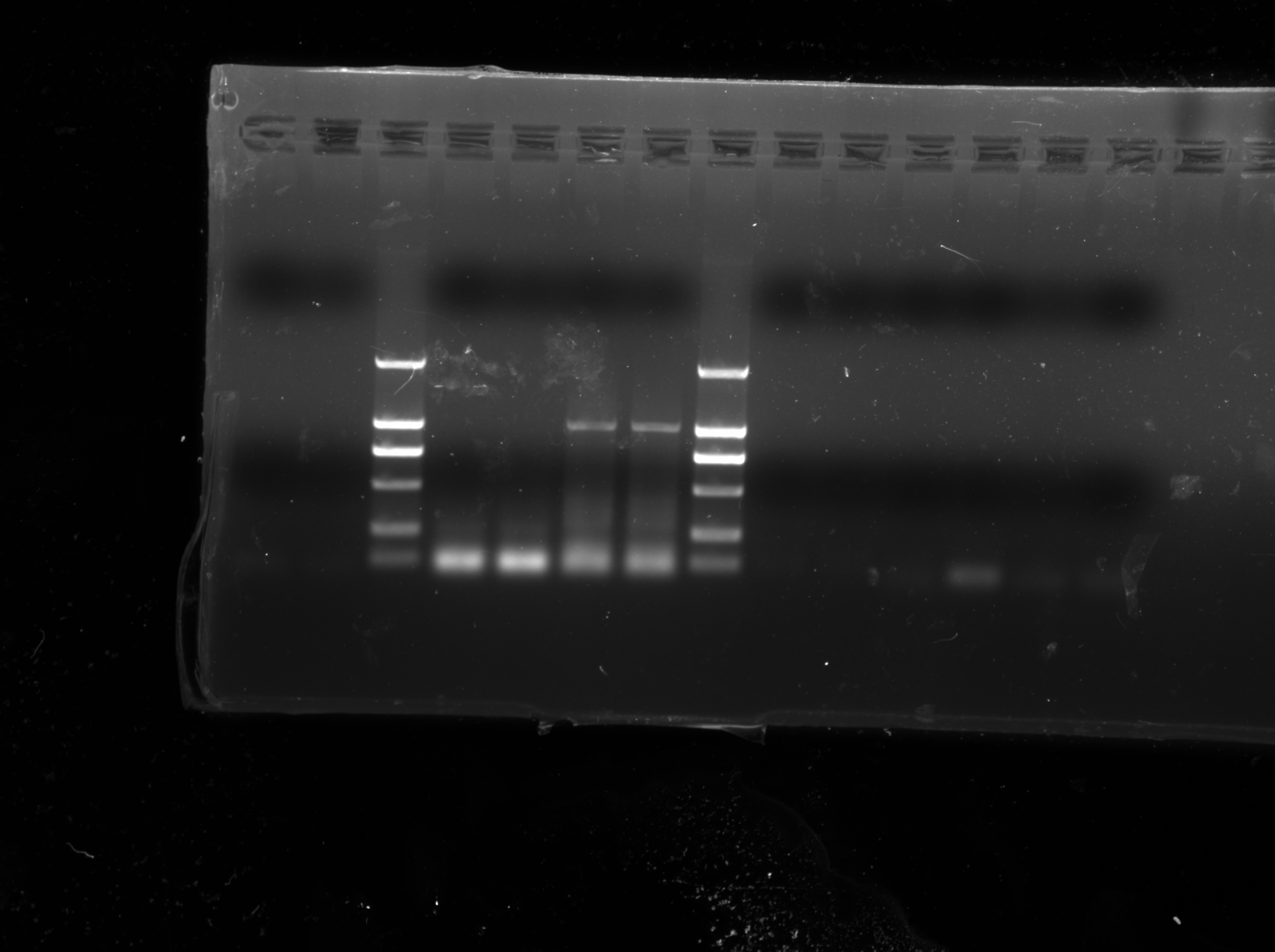

Supplement: Supplemental Information 4 [file peerj-11-15045-s004.zip › Figure 1C.png]

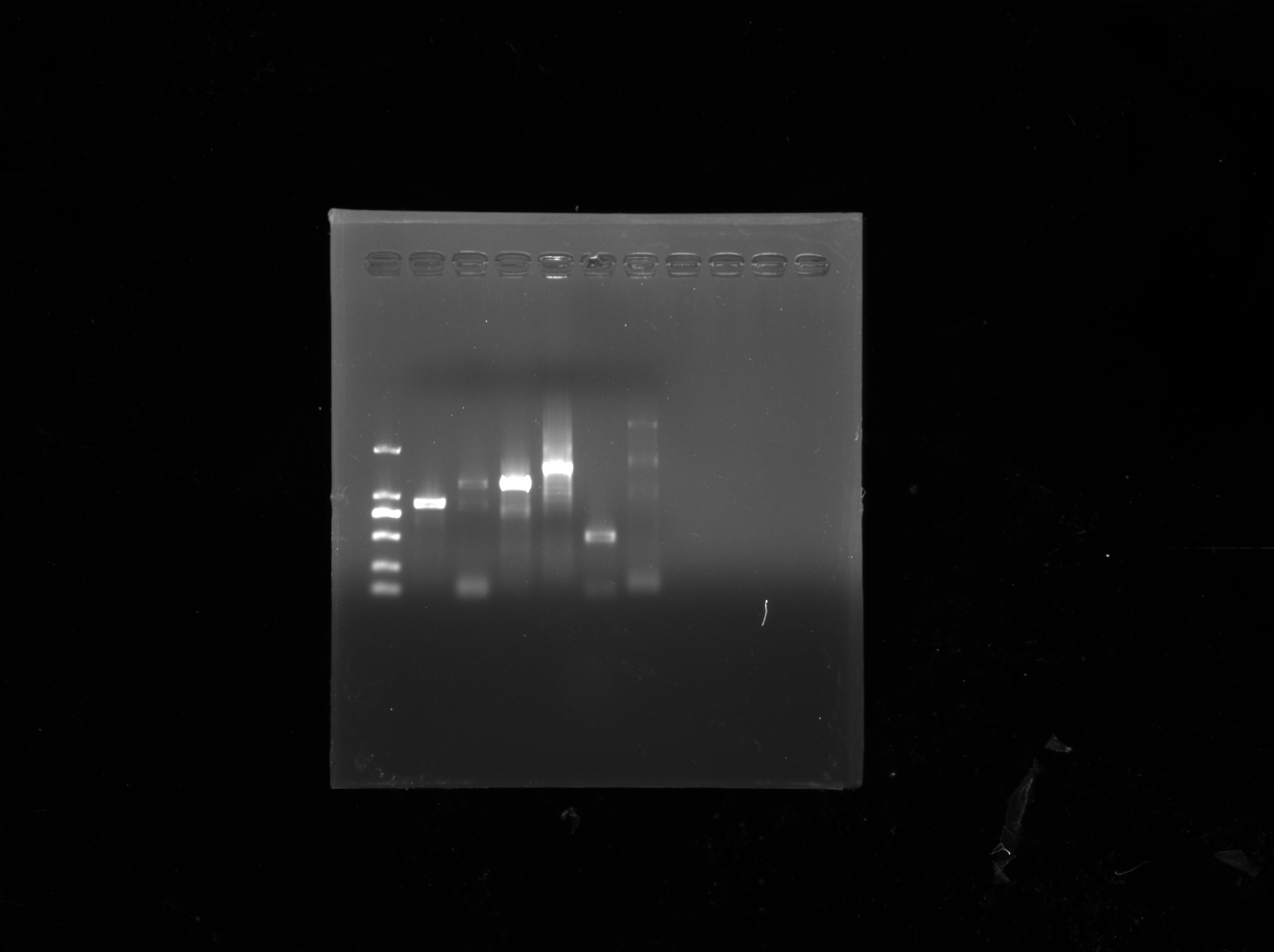

Supplement: Supplemental Information 4 [file peerj-11-15045-s004.zip › Figure 1D.png]

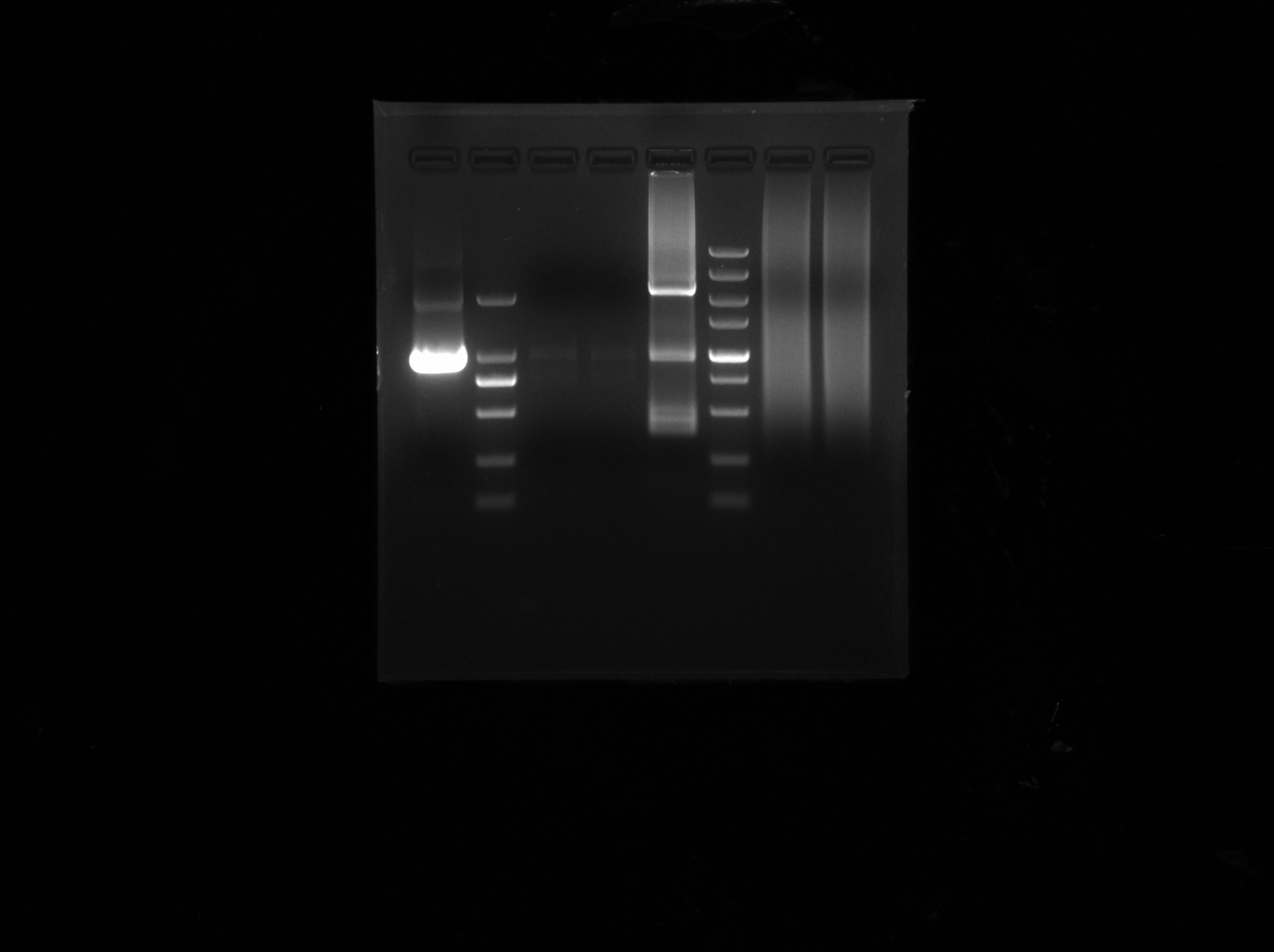

Supplement: Supplemental Information 4 [file peerj-11-15045-s004.zip › Figure 1E.png]
