# Supplementary material for: Cloning, characterization, and expression analysis of the CHITINASE gene family in Helice tientsinensis
Source: PeerJ. 2023 Mar 14;11:e15045. doi: 10.7717/peerj.15045 (PMC10022498; doi:10.7717/peerj.15045)
Supplement: Supplemental Information 5 [file peerj-11-15045-s005.docx]

**Supplemental Table S1：**

**Accession numbers of NCBI Genbank used in phylogenetic analysis.**

| **Group** | **Gene name** | **Accession number** |
| --- | --- | --- |
| Group Ⅲ | *Macrobrachium_nipponense_CHT3A* | KF466276.1 |
| Group Ⅲ | *Palaemon_carinicauda_CHT4* | MF173615.1 |
| Group Ⅲ | *Macrobrachium_nipponense_CHT3C* | KF466278.1 |
| Group Ⅲ | *Palaemon_carinicauda_CHT2* | MF173613.1 |
| Group Ⅲ | *Pandalopsis_japonica_CHT4* | JF694839.1 |
| Group Ⅲ | *Pandalopsis_japonica_CHT3* | JF694838.1 |
| Group Ⅲ | *Macrobrachium_rosenbergii_CHT3* | LT574899.1 |
| Group Ⅲ | *Palaemon_carinicauda_CHT* | MF173612.1 |
| Group Ⅲ | *Pandalopsis_japonica_CHT2* | JF694837.1 |
| Group Ⅲ | *Macrobrachium_nipponense_CHT3* | KF887954.1 |
| Group Ⅲ | *Macrobrachium_nipponense_CHT3B* | KF466277.1 |
| Group Ⅲ | *Eriocheir_sinensis_CHT3* | KP692740.1 |
| Group Ⅲ | ***Helice_tientsinensis_CHT3*** |  |
| Group Ⅲ | *Portunus_trituberculatus_CHT3* | AB874469.1 |
| Group Ⅲ | *Scylla_paramamosain_CHT3* | MF536873.1 |
| Group Ⅲ | *Scylla_serrata_CHT* | EU883590.1 |
| Group Ⅲ | *Penaeus_monodon_CHT3* | GU344707.1 |
| Group Ⅲ | *Fenneropenaeus_chinensis_CHT* | DQ277710.1 |
| Group Ⅲ | *Litopenaeus_vannamei_CHT3* | EU381118.1 |
| Group Ⅲ | *Marsupenaeus_japonicus_CHT3* | AB008027.1 |
| Group Ⅴ | *Scylla_paramamosain_CHT5partial* | MF536875.1 |
| Group Ⅴ | *Portunus_trituberculatus_CHT5* | MH160827.1 |
| Group Ⅴ | *Macrophthalmus_japonicus_CHT5* | KP219427.1 |
| Group Ⅴ | *Litopenaeus_vannamei_CHT4* | FJ888480.1 |
| Group Ⅴ | *Litopenaeus_vannamei_CHT5* | FJ888481.1 |
| Group Ⅴ | *Penaeus_monodon_CHT5* | KY865407.1 |
| Group Ⅳ | *Eriocheir_sinensis_CHT4* | KP692741.1 |
| Group Ⅳ | ***Helice_tientsinensis_CHT4*** |  |
| Group Ⅳ | *Macrophthalmus_japonicus_CHT4partial* | KP219426.1 |
| Group Ⅳ | *Scylla_paramamosain_CHT4* | MF536874.1 |
| Group Ⅳ | *Portunus_trituberculatus_CHT4* | MH589127.1 |
| Group Ⅳ | *Penaeus_monodon_CHT4* | KY210138.1 |
| Group Ⅳ | *Pandalopsis_japonica_CHT5* | JF694840.1 |
| Group Ⅳ | *Macrobrachium_nipponense_CHT4* | KF466279.1 |
| Group Ⅵ | *Scylla_paramamosain_CHT6* | MF536876.1 |
| Group Ⅵ | *Portunus_trituberculatus_CHT6* | MH137202.1 |
| Group Ⅵ | *Eriocheir_sinensis_CHT6* | KP692742.1 |
| Group Ⅵ | *Litopenaeus_vannamei_CHT6* | GQ916594.1 |
| Group Ⅱ | *Portunus_trituberculatus_CHT2* | KM100755.1 |
| Group Ⅱ | *Scylla_serrata_CHT2* | GU168777.1 |
| Group Ⅱ | *Eriocheir_sinensis_CHT2* | KP692739.1 |
| Group Ⅱ | *Litopenaeus_vannamei_CHT2paetial* | FJ888479.1 |
| Group Ⅱ | *Penaeus_monodon_CHT2* | KX610684.1 |
| Group Ⅱ | *Marsupenaeus_japonicus_CHT2* | D89751.1 |
| Group Ⅰ | *Macrobrachium_nipponense_CHT1* | KF963281.1 |
| Group Ⅰ | *Macrobrachium_nipponense_CHT1C* | KF963281.1 |
| Group Ⅰ | *Macrobrachium_nipponense_CHT1A* | KF466274.1 |
| Group Ⅰ | *Macrobrachium_nipponense_CHT1B* | KF466275.1 |
| Group Ⅰ | *Palaemon_carinicauda_CHT1* | MF173612.1 |
| Group Ⅰ | *Litopenaeus_vannamei_CHT1* | EU883591.1 |
| Group Ⅰ | *Fenneropenaeus_chinensis_CHT1* | DQ277710.1 |
| Group Ⅰ | *Penaeus_monodon_CHT1* | AF157503.1 |
| Group Ⅰ | *Marsupenaeus_japonicus_CHT1* | D84250.1 |
| Group Ⅰ | *Scylla_paramamosain_CHT1* | MF536871.1 |
| Group Ⅰ | *Scylla_serrata_CHT1* | EU883590.1 |
| Group Ⅰ | *Portunus_trituberculatus_CHT1* | KM100752.1 |
| Group Ⅰ | *Macrophthalmus_japonicus_CHT1partial* | KP219425.1 |
| Group Ⅰ | *Eriocheir_sinensis_CHT1* | KP692738.1 |
| Group Ⅰ | ***Helice_tientsinensis_CHT1*** |  |
| Group Ⅰ | *Pandalopsis_japonica_CHT1* | JF694836.1 |
